# *piv* does not impact *Pseudomonas aeruginosa* virulence in *Galleria mellonella*

**DOI:** 10.1128/spectrum.02811-24

**Published:** 2025-05-21

**Authors:** Rachel E. Robinson, Joshua K. Robertson, Dina A. Moustafa, Joanna B. Goldberg

**Affiliations:** 1 Microbiology and Molecular Genetics Program, Graduate Division of Biological and Biomedical Sciences, Laney Graduate School, Emory University 1371, Atlanta, Georgia, USA; 2 Department of Pediatrics, Division of Pulmonary, Asthma, Cystic Fibrosis, and Sleep, Emory University School of Medicine 12239, Atlanta, Georgia, USA; 3 Department of Biology, Emory University 1371, Atlanta, Georgia, USA; 4 Emory+Children’s Center for Cystic Fibrosis and Airway Disease Research, Emory University School of Medicine 12239, Atlanta, Georgia, USA; Università Roma Tre, Rome, Italy

**Keywords:** *Pseudomonas aeruginosa*, temperature regulation, proteases, *Galleria*, virulence factors

## Abstract

*Pseudomonas aeruginosa* is an opportunistic human pathogen that can also infect mammals, invertebrates, and plants. Protease IV (PIV) is a secreted protease shown to be important in mammalian cornea, lung, and wound models of infection. It also contributes to *P. aeruginosa* virulence in many invertebrate models. Previous studies have shown that the expression of the gene encoding PIV is higher at 25°C than at 37°C. Thus, we hypothesized that *piv* would be more important for *P. aeruginosa* virulence at 25°C than at 37°C. To test this, we first demonstrated that more PIV is secreted by *P. aeruginosa* PAO1 cells grown at 25°C than at 37°C. We then determined the survival of larvae of the greater wax moth *Galleria mellonella* infected by PAO1 and an isogenic Δ*piv* mutant at both 25°C and 37°C. We found no significant difference in virulence between PAO1 and Δ*piv* at either 25°C or 37°C, although both strains were more virulent at 37°C than 25°C as measured by a decrease in median survival time. *P. aeruginosa* possesses an arsenal of virulence factors besides PIV, and thus loss of this single virulence factor may not result in attenuation in the highly susceptible *G. mellonella* larvae.

**IMPORTANCE:**

Pathogenesis of the important opportunistic pathogen *Pseudomonas aeruginosa* is often investigated using model organisms. Larvae of the greater wax moth, *Galleria mellonella*, are a popular non-mammalian model organism for *P. aeruginosa* infections that have been used to study highly attenuated mutants and characterize their defects in virulence. Our study shows that small differences in the virulence of *P. aeruginosa*, such as those caused by deleting the gene encoding a single virulence factor, may not be detectable in the *G. mellonella* model of infection. This is an important finding for researchers considering the choice of model organisms for virulence studies.

## INTRODUCTION

The opportunistic pathogen *Pseudomonas aeruginosa* produces many virulence factors that enable it to cause infections. Virulence factors are commonly identified by strains mutated in the gene(s) of interest being attenuated in a model organism. Protease IV (PIV, also called PrpL for PvdS-regulated endoprotease, lysyl class [1]) is a secreted protease that has been shown to contribute to *P. aeruginosa* virulence in multiple models of infection. PIV is a serine protease that cleaves on the carboxyl side of lysine residues with optimum catalytic activity at 45°C and pH 10 (2). The catalytic activity of PIV increases from 10°C to 45°C and decreases thereafter, presumably due to the thermal liability of the protein (2). PIV causes epithelial erosion and damage in corneal infections, and mutants lacking *piv* are less virulent in both rabbit and murine models of corneal infection (3–5). Administration of purified PIV to wounds is sufficient to delay healing; *piv* mutants have been shown to cause less severe wound infections that heal faster and are also attenuated in murine acute lung infections (6, 7). Purified PIV has been shown to degrade alveolar surfactant proteins, which opsonize bacteria to promote clearance by alveolar macrophages, as well as the cytokine IL-22, which is important for the lung epithelial immune response to bacterial infection, both *in vitro* and *in vivo* (8–10). Many other components of the innate immune response have been shown to be degraded *in vitro* by PIV, including the iron-sequestering proteins lactoferrin and transferrin, and complement component 3 (1, 2).

Previous studies have shown that *piv* expression is higher at ambient temperatures of 22°C–28°C than 37°C (11, 12), which we have recently elucidated is due to the upregulation of *piv* by the quorum sensing transcriptional regulator LasR more at 25°C than 37°C (13). Given the thermoregulation of this virulence factor, we wondered what the importance of *piv* would be to *P. aeruginosa* virulence at different temperatures. We chose to test this using the *Galleria mellonella* (greater wax moth) larvae model of infection for several reasons. *G. mellonella* is an inexpensive model organism that is generally easy to manipulate and does not require specialized equipment, animal care facilities, or approval by an Institutional Animal Care and Use Committee (IACUC). Like other insects, *G. mellonella* larvae possess an innate immune system with features similar to the mammalian immune system, including hemocyte activity (comparable to neutrophils) as well as opsonin and antimicrobial peptide production (14). PIV has been shown to degrade apolipophorin-III, an important opsonin of the innate immune system, in *in vitro* assays using hemolymph derived from *G. mellonella* (14, 15). During *P. aeruginosa* infection, apolipophorin-III is degraded into products similar to those produced by PIV activity *in vitro*, suggesting that PIV may contribute to apolipophorin-III degradation *in vivo* (15, 16). Many virulence factors important in *G. mellonella* infections are also important for successful murine infection, suggesting that *G. mellonella* is a good proxy for mouse infections (and by extension human infections) of *P. aeruginosa,* and thus it has been well studied (17–20). Crucially, *G. mellonella* larvae can be incubated at 25°C and 37°C to manipulate the host temperature (21), which provides an opportunity to study how temperature and thermoregulation impact the virulence of *P. aeruginosa*. The *G. mellonella* larvae model has been previously used to study temperature-dependent virulence factors in *P. aeruginosa* and other bacterial pathogens (20, 22).

Because *piv* expression is higher at 25°C than at 37°C, we hypothesized that *piv* would be more important for *P. aeruginosa* virulence at 25°C compared to 37°C. To test this hypothesis, we demonstrated that more PIV is secreted by *P. aeruginosa* PAO1 grown at 25°C than at 37°C, in accordance with how *piv* expression is thermoregulated. We then generated an isogenic, clean deletion mutant of *piv* in PAO1, referred to as Δ*piv*, to test its role in killing *G. mellonella* larvae at different temperatures. Surprisingly, we found that PAO1 and Δ*piv* are equally virulent in a *G. mellonella* killing assay at both 25°C and 37°C. We believe this is not because PIV is unimportant for virulence, but rather because both PAO1 and Δ*piv* possess numerous other virulence factors that are sufficient to overwhelm the highly susceptible *G. mellonella* larvae.

## MATERIALS AND METHODS

### Culture conditions, bacterial strains, and plasmids

*P. aeruginosa* was routinely cultured overnight in 3 mL of lysogeny broth (LB) at 37°C in test tubes on a roller drum. Bacteria were subcultured at either 37°C or 25°C in flasks, as indicated, shaking at 200 rpm. *Escherichia coli* growth media was supplemented with carbenicillin (100 µg/mL), and *P. aeruginosa* media with carbenicillin (300 µg/ml) and/or tetracycline (100 µg/ml) as needed. All strains used in this study are listed in Table 1.

**TABLE 1:** Bacterial strains used in this study

Strain	Source
*Escherichia coli* 5-alpha (DH5α derivative)	New England Biolabs (NEB)
*Pseudomonas aeruginosa* PAO1	Simon Dove (Harvard University)
PAO1 PIV VSV-G	Robinson et al. (13)
PAO1 Δ*piv*	This study

PAO1 Δ*piv* was constructed using a CRISPR-Cas9 system (23) to create deletions, with minor modifications as previously described (13). Briefly, PAO1 carrying pCasPA was electroporated with pRD83 (described below) to delete *piv*. The Δ*piv* mutant was confirmed by PCR with primers oRD41/oRD42 and sequencing (Azenta Life Sciences, Chelmsford, MA, USA).

pRD83 was constructed by isothermal assembly (24) with Gibson Assembly Master Mix (New England Biolabs [NEB], Ipswich, MA, USA) according to the manufacturer’s instructions. Primers used in this study are listed in Table 2. The spacer for *piv* composed of annealed primers oRD149/oRD150 was inserted into BsaI-digested pACRISPR by Golden Gate assembly (25). The PCR product of oRD172/oRD173 and oRD174/oRD175, both amplified from PAO1 genomic DNA, was assembled into the *piv* spacer-containing intermediate pACRISPR plasmid digested with XbaI and XhoI before transformation by heat shock into competent *Escherichia coli* 5-alpha (NEB) according to the manufacturer’s instructions. pRD83 was confirmed by sequencing (Plasmidsaurus, Eugene, OR, USA).

**TABLE 2:** Primers used in this study

Primer	Sequence
oRD15	cctgctgaacaacggcaac
oRD16	agcactgggtggtgttgtag
oRD41	tgcactacatcctgcacctc
oRD42	gggataaacggcggataacac
oRD149	gtggtcccgctacttcgcgccctg
oRD150	aaaccagggcgcgaagtagcggga
oRD172	cgagtcggtgctttttttgagatctgtccatacccatggtagtaatggtaagcggccag
oRD173	gccggtgcgcgatcgatagcctggctggccgaatcgactccttcagttt
oRD174	ggccagccaggctatcga
oRD175	agaatactcaagcttctgaatggcgggagtatgaaaagtcattcctcctgcccctccg
oRD191	gcacaacatcatcgccatcc
oRD192	tcgacgacttcaccgttgtt

### Supernatant concentration and protein precipitation

Overnight biological triplicates of PAO1 PIV VSV-G were subcultured to an initial OD_600_ of 0.05 in 25 mL LB and incubated shaking at 200 rpm at 25°C and 37°C. At early stationary phase (OD_600_ = 2.0), cells were centrifuged at 13,000 x *g* for 10 min at 25°C. The resulting supernatant was sterilized using a 0.22 µM filter to remove any remaining cells and subsequently concentrated using a Pierce Protein Concentrator PES 3K MWCO (ThermoFisher) according to the manufacturer’s guidance. Supernatants were concentrated to approximately 20× to 1 mL final volume. Trichloroacetic acid was added to a final concentration of 20%, samples incubated overnight at 4°C, and then centrifuged at 21,000 x *g* for 30 min at 4°C. Pellets were washed three times with cold acetone and allowed to dry completely at 99°C before resuspending in Laemmli buffer containing β-mercaptoethanol. The pH of the resuspension was adjusted with 1 M NaOH as needed and boiled for 30 min prior to immunoblotting as previously described (13).

#### *G. mellonella* infections

*G. mellonella* larvae (SpeedyWorm, Alexandria, MN, USA) were housed and infected as previously described (18) with minor modifications as follows. Overnight biological triplicates of PAO1 and Δ*piv* were subcultured to an initial OD_600_ of 0.05 in 25 mL LB and incubated shaking at 200 rpm at 25°C and 37°C until exponential phase (OD_600_ = 0.5). *G. mellonella* larvae weighing between 150 and 200 mg were injected with ~100 colony-forming units (CFU) or ~10 CFU as indicated in 5 µL phosphate-buffered saline (PBS) or an equal volume of PBS as an injection control and incubated at the corresponding temperature for up to 48 h in a petri dish. Larvae were monitored for melanization and activity, and larval death was determined by a lack of response to stimulus and complete blackening. The results were analyzed with Kaplan-Meier survival curves and log-rank tests using GraphPad Prism version 10.

### RNA extraction and real-time quantitative PCR (RT-qPCR)

*G. mellonella* larvae were infected with ~100 CFU of PAO1 and Δ*piv* as described above. At 16 h post-infection for the 37°C groups and 40 h post-infection for the 25°C groups, five larvae per group were anesthetized on ice, and hemolymph was collected by cutting off the tails. Hemolymph from larvae of the same group was pooled and centrifuged at 5,000 g to remove cell debris, and RNA was extracted from the subsequent cell-free hemolymph using Tri-Reagent (Millipore Sigma) according to the manufacturer’s instructions. RNA was then treated with TURBO DNase (ThermoFisher) using the manufacturer’s instructions for rigorous DNase treatment. RT-qPCR was conducted using Power SYBR™ Green RNA-to-CT™ 1-Step Kit (Applied Biosystems) with primers oRD15/oRD16 for *piv* and primers oRD191/oRD192 for *mvaU* on a LightCycler 96 (Roche) using LightCycler software v1.1.0.1320 in technical triplicate. Triplicate reactions were pooled and analyzed by gel electrophoresis. RT-qPCR primers are designed to yield products of approximately 100 bp.

## RESULTS AND DISCUSSION

Our lab had previously found that *piv* gene expression is thermoregulated at the level of transcription, with significantly higher expression at ambient temperatures such as 25°C than at 37°C (13). We wondered how temperature affects the amount of PIV protease produced and how this might affect the virulence of PAO1 at 25°C versus 37°C.

**Fig 1:**
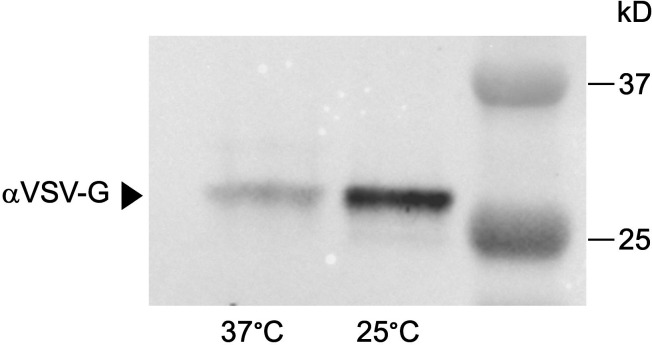
Mature PIV VSV-G protein levels in *P. aeruginosa* supernatants are thermoregulated. Supernatant harvested from PAO1 PIV VSV-G grown to the early stationary phase at 25°C and 37°C was concentrated, and total proteins were precipitated prior to analysis by immunoblotting with antibodies against the VSV-G tag (αVSV-G). A representative image of three biological replicates is shown. Mature PIV VSV-G found in supernatant is approximately 28 kD.

First, we wanted to confirm how temperature affects the amount of PIV protease present in the supernatant of cells grown at 25°C compared to 37°C, as secreted PIV would interact with the host during a *P. aeruginosa* infection. To do this, we used a strain of PAO1 in which the chromosomal *piv* gene was tagged at the C-terminus with the vesicular stomatitis virus G (VSV-G) epitope (13). As the PIV precursor protein is post-translationally modified during secretion, the enzymatically active (“mature”) form of PIV found in the supernatant is 26 kD (2, 26); the addition of the VSV-G tag results in a mature PIV VSV-G protein that is approximately 28 kD. The PAO1 PIV VSV-G strain was grown at 25°C and 37°C to the stationary phase, supernatant from an equal number of cells harvested, and total secreted proteins precipitated prior to western blotting with VSV-G antibodies (αVSV-G). As expected, we found more mature PIV in the supernatant harvested from cells grown at 25°C than at 37°C (Fig. 1).

**Fig 2:**
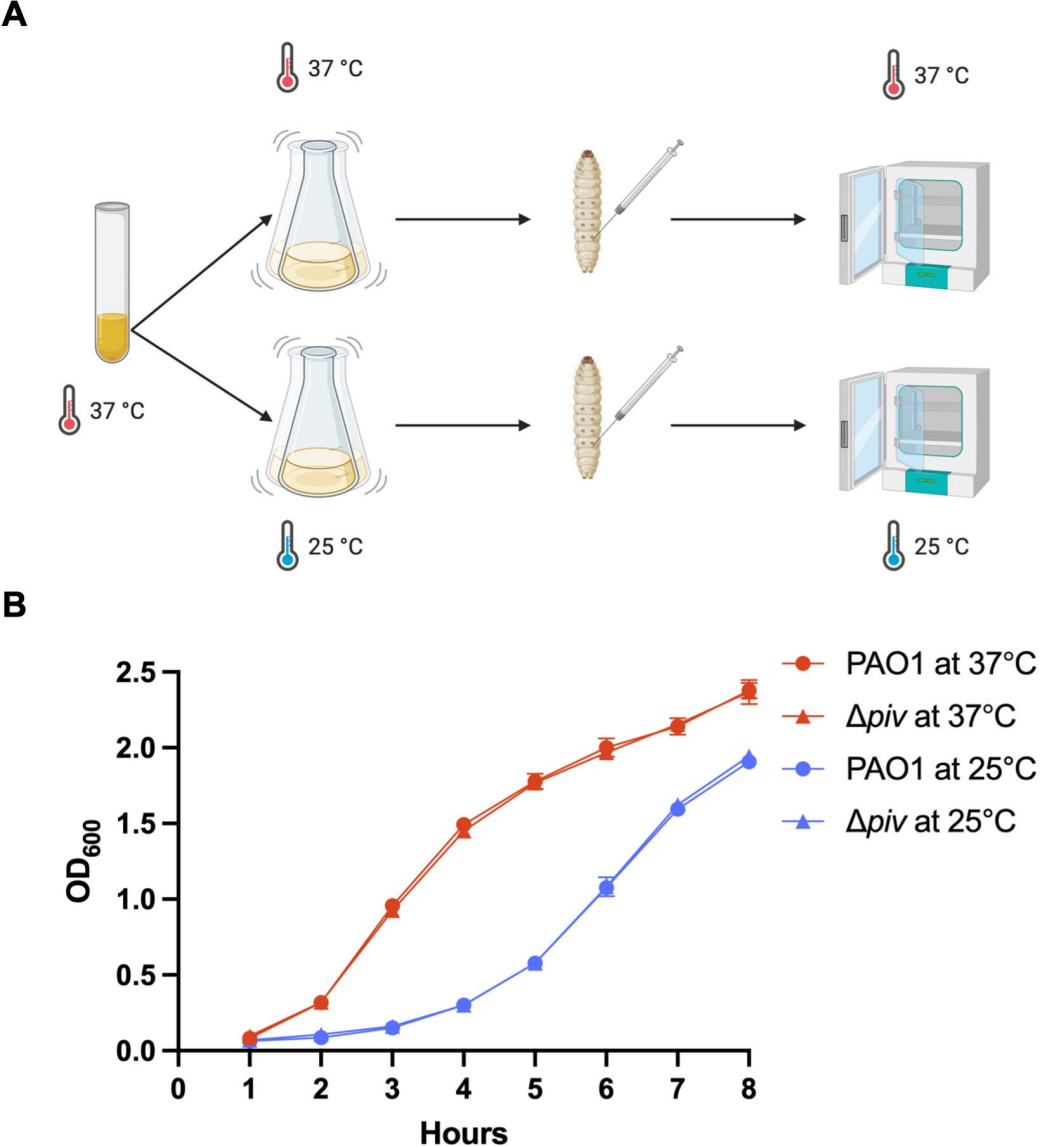
Schematic of *G. mellonella* larvae infection by *P. aeruginosa* strains at two temperatures. (**A**) Overnight cultures of PAO1 and Δ*piv* grown at 37°C were diluted to an OD_600_ = 0.05 and cultured at 37°C and 25°C until reaching exponential phase at OD_600_ approximately 0.5. *G. mellonella* larvae were infected with 100 CFU of each strain grown at each temperature and then incubated at the corresponding temperature to assess the role of *piv* in *P. aeruginosa* virulence at 37°C compared to 25°C. Created with BioRender.com. (**B**) PAO1 and Δ*piv* were grown overnight at 37°C and subcultured at 37°C and 25°C. The optical density (OD_600_) of cultures was measured hourly for 8 h. Three biological replicates are shown with error bars representing standard deviation.

**Fig 3:**
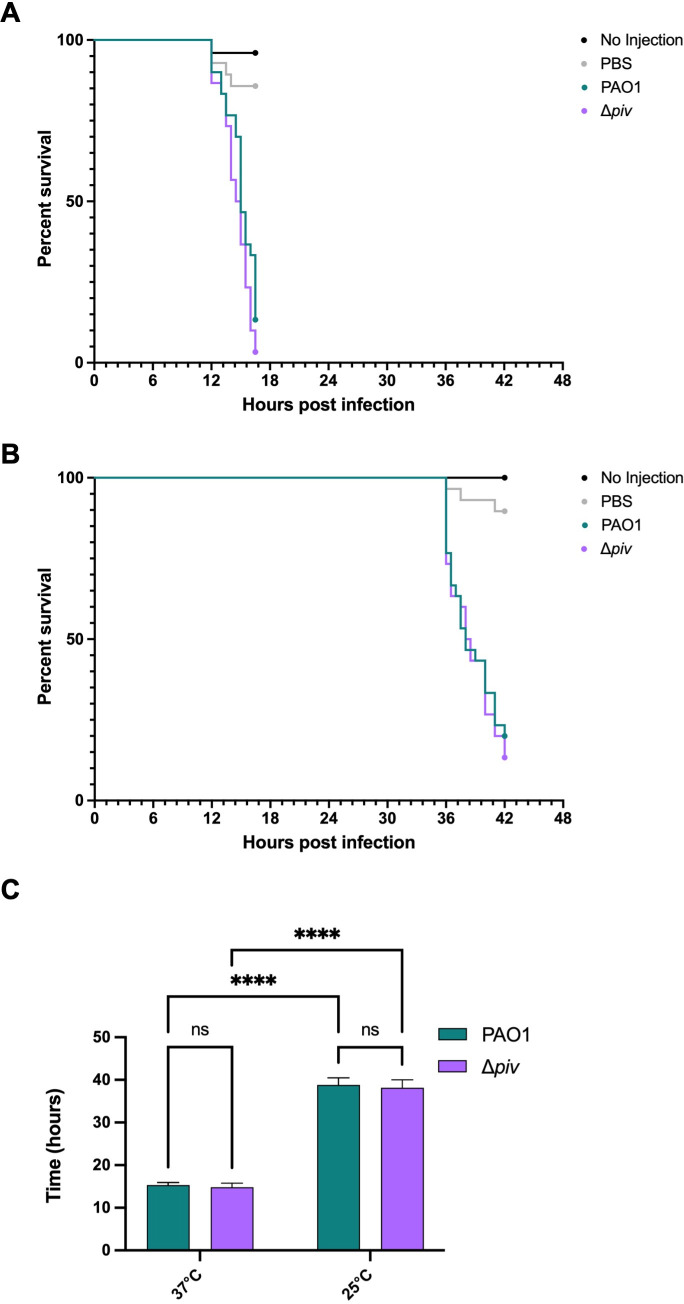
Deletion of *piv* does not impact the virulence of *P. aeruginosa* in *G. mellonella. G. mellonella* larvae were infected with ~100 CFU of PAO1 and Δ*piv* from exponential phase cultures grown at 37°C (**A**) or 25°C (**B**) and then housed at the corresponding temperature as depicted in Fig. 2. Larvae were also injected with an equal volume of PBS or not injected as negative controls. Thirty larvae were infected for each PAO1 and Δ*piv* group, 28 for the PBS group, and 25 for the no-infection group at each temperature. Kaplan-Meier survival curves represent the combined data from three independent experiments. Statistical significance was determined by the log-rank test. (**C**) Median survival for larvae infected with PAO1 and Δ*piv* at both 37°C and 25°C shown in (A) and (B). The mean of the three independent experiments with standard deviation is shown. Statistical significance was determined by two-way ANOVA with uncorrected Fisher’s LSD test: ns—not significant and ****P < 0.0001.

To test how thermoregulation of *piv* would affect the virulence of PAO1 to *G. mellonella* at 25°C compared to 37°C, we infected *G. mellonella* larvae with ~100 CFU of PAO1 and Δ*piv* grown at 25°C and 37°C and then housed the larvae at the corresponding temperature (Fig. 2A). Δ*piv* was not defective in growth *in vitro* compared to PAO1 at either 37°C or 25°C (Fig. 2B). We found no significant difference (*P* > 0.0332) in mortality between larvae infected with PAO1 and Δ*piv* at either 37°C (4/30 and 1/30, surviving, respectively, Fig. 3A) or 25°C (6/30 and 4/30 surviving, respectively, Fig. 3B). We note that the median time to death for larvae infected by PAO1 in the 25°C infection condition (38 h) was significantly longer (*P* < 0.0001) than in the 37°C condition (15 h), and similar for larvae infected by Δ*piv*, indicating that virulence of *P. aeruginosa* in this model system is affected by temperature (Fig. 3C). In a previous study in which *P. aeruginosa* was used to infect *G. mellonella* at both 25°C and 37°C, similar patterns of temperature-dependent virulence with longer survival times at 25°C were observed (20).

We wondered if the lack of difference in virulence between PAO1 and Δ*piv* could be due to *piv* not being expressed during infection of *G. mellonella*. To test this, we again infected *G. mellonella* larvae with ~100 CFU of PAO1 and Δ*piv* at both 37°C and 25°C as diagrammed in Fig. 2A and collected hemolymph at 16 h post-infection for 37°C groups and at 40 h post-infection for 25°C groups. RNA was extracted from hemolymph and used in RT-qPCR with primers to detect expression of *piv* and *mvaU*, a control gene that does not vary with temperature (13), during infection of *G. mellonella* by PAO1 and Δ*piv* at both 37°C and 25°C (Fig. S1). We found that *piv* was expressed by PAO1 during *G. mellonella* infection at both 37°C and 25°C; thus, the lack of difference in the virulence between PAO1 and Δ*piv* in this model is not due to the lack of expression of *piv*.

We also tested if a lower inoculum could reveal a greater difference in virulence between the strains and repeated the infection of *G. mellonella* larvae with PAO1 and Δ*piv* at both 37°C and 25°C with ~10 CFU of each strain (Fig. S2). We again found no significant difference (*P* > 0.0332) in mortality between larvae infected with ~10 CFU of PAO1 and Δ*piv* at 37°C (Fig. S2A). At 25°C (Fig. S2B), there was a slight difference in mortality at earlier time points of the infection (*P* < 0.0021), but comparable numbers of larvae infected with both PAO1 and Δ*piv* succumbed to infection by 40 h (0/40 and 4/40 larvae surviving, respectively). Ultimately, inoculum doses as low as ~10 CFU did not reveal a great difference in virulence between PAO1 and Δ*piv* strains.

We were surprised to find a negligible difference in virulence between PAO1 and Δ*piv* in this *G. mellonella* larvae model of infection (Fig. 3; Fig. S2),given that PIV has been shown to contribute to virulence in other invertebrate models of infection, including *T. molitor* larvae*, Caenorhabditis elegans*, and *Artemia salina* (brine shrimp) when these organisms were infected with PAO1 and Δ*piv* cells (7). PIV was also shown to contribute to virulence in *Tenebrio molitor* larvae when larvae were injected with cell-free supernatants of strains overexpressing and/or lacking PIV (27, 28).

*P. aeruginosa* is highly virulent to *G. mellonella* when the bacteria are administered via injection (17, 19, 29). It is possible that deleting only *piv*, a single secreted virulence factor, did not significantly diminish the overall virulence of the bacterium in this highly susceptible insect host. In support of this, we note that *G. mellonella* larvae infected with as few as 10 CFU of PAO1 or Δ*piv* strains do not survive longer than those infected with 100 CFU of the same strain, underscoring how virulent *P. aeruginosa* is in this model organism. *P. aeruginosa* possesses an arsenal of both secreted and contact-dependent virulence factors. One contact-dependent virulence factor, the type III secretion system (TTSS), has been shown to contribute significantly to the virulence of the strain PA14 in *G. mellonella* (19). In our study, the Δ*piv* strain did not differ from the parent strain in terms of its TTSS, which would be consistent with the TTSS being a major virulence factor in this model system and our finding that the deletion of *piv* did not impact virulence. Supporting this, previous studies have shown that the loss of only one of the major TTSS effectors did not significantly alter *P. aeruginosa* virulence in *G. mellonella* and that only the loss of two or more effectors resulted in attenuation (19). Consistent with this previous study and our current results, a study of PAO1 infections of *G. mellonella* found that single mutants deficient in the pilus, flagella, or elastase production were also not less virulent than the wildtype strain, even though these factors are known to be involved in *P. aeruginosa* virulence in many other model systems (29). We suspect that if thermoregulation of PIV was studied in a less virulent *P. aeruginosa* strain, an effect might be discernible.

Differences in the *G. mellonella* host versus other invertebrates tested for *P. aeruginosa* infections could also explain why we did not find a difference in virulence between Δ*piv* and PAO1 strains. As previously mentioned, *piv* mutant strains are attenuated in *C. elegans* and other invertebrates, which differs from our findings in *G. mellonella*. There could be host factors of *G. mellonella* that target the secreted PIV protease to diminish its proteolytic activity. Furthermore, although PIV was shown to degrade apolipophorin-III in hemolymph derived from *G. mellonella*, it is unknown the extent to which degradation of apolipophorin-III would contribute to larval mortality. Interestingly, the TTSS, which is important in *G. mellonella* larvae infections, was not found to contribute to *P. aeruginosa* virulence in *C. elegans* (19). This indicates that within invertebrates, there are organism-dependent differences in *P. aeruginosa* pathogenesis and could explain why *piv* appears less critical to *P. aeruginosa* virulence in *G. mellonella* when compared to other species previously tested.

In conclusion we found that *piv* is not a crucial virulence factor for *P. aeruginosa* to cause infections in *G. mellonella*; however, we did find that temperature affected the virulence of both PAO1 and Δ*piv* strains with increased virulence at 37°C compared to 25°C. As Δ*piv* mutants have been shown to be attenuated in other invertebrates (7, 28), our work highlights the importance of selecting an appropriate model for assessing bacterial virulence. Temperature is important in the transition of a pathogen from the environment to the host, and *G. mellonella* is a unique model organism that allows for the role of thermoregulation in virulence to be tested. However, the sensitivity of *G. mellonella* to *P. aeruginosa* may not be ideal when small changes in virulence are studied due to an inability to discern the effect on the survival of the *G. mellonella* larvae, as we have shown. This underscores the importance of the choice of model organisms in pathogenesis studies.

## SUPPLEMENTAL MATERIAL

Supplemental material spectrum.02811-24-s0001.docx Figures S1 and S2.

ASM does not own the copyrights to Supplemental Material that may be linked to, or accessed through, an article. The authors have granted ASM a non-exclusive, world-wide license to publish the Supplemental Material files. Please contact the corresponding author directly for reuse.

## References

[1] Wilderman PJ, Vasil AI, Johnson Z, Wilson MJ, Cunliffe HE, Lamont IL, Vasil ML. 2001. Characterization of an endoprotease (PrpL) encoded by a PvdS-regulated gene in Pseudomonas aeruginosa. Infect Immun 69:5385–5394. doi:10.1128/IAI.69.9.5385-5394.2001 11500408 PMC98648

[2] Engel LS, Hill JM, Caballero AR, Green LC, O’Callaghan RJ. 1998. Protease IV, a unique extracellular protease and virulence factor from Pseudomonas aeruginosa. J Biol Chem 273:16792–16797. doi:10.1074/jbc.273.27.16792 9642237

[3] Engel LS, Hobden JA, Moreau JM, Callegan MC, Hill JM, O’Callaghan RJ. 1997. Pseudomonas deficient in protease IV has significantly reduced corneal virulence. Invest Ophthalmol Vis Sci 38:1535–1542. 9224281

[4] Engel LS, Hill JM, Moreau JM, Green LC, Hobden JA, O’Callaghan RJ. 1998. Pseudomonas aeruginosa protease IV produces corneal damage and contributes to bacterial virulence. Invest Ophthalmol Vis Sci 39:662–665. 9501882

[5] O’Callaghan RJ, Engel LS, Hobden JA, Callegan MC, Green LC, Hill JM. 1996. Pseudomonas keratitis. the role of an uncharacterized exoprotein, protease IV, in corneal virulence. Invest Ophthalmol Vis Sci 37:534–543. 8595953

[6] Prasad ASB, Shruptha P, Prabhu V, Srujan C, Nayak UY, Anuradha CKR, Ramachandra L, Keerthana P, Joshi MB, Murali TS, Satyamoorthy K. 2020. Pseudomonas aeruginosa virulence proteins pseudolysin and protease IV impede cutaneous wound healing. Lab Invest 100:1532–1550. doi:10.1038/s41374-020-00478-1 32801335 PMC7683349

[7] Kim T-H, Li X-H, Lee J-H. 2021. Alleviation of Pseudomonas aeruginosa infection by propeptide-mediated inhibition of protease IV. Microbiol Spectr 9:e0078221. doi:10.1128/Spectrum.00782-21 34704789 PMC8549743

[8] Guillon A, Brea D, Morello E, Tang A, Jouan Y, Ramphal R, Korkmaz B, Perez-Cruz M, Trottein F, O’Callaghan RJ, Gosset P, Si-Tahar M. 2017. Pseudomonas aeruginosa proteolytically alters the interleukin 22-dependent lung mucosal defense. Virulence 8:810–820. doi:10.1080/21505594.2016.1253658 27792459 PMC5626239

[9] Malloy JL, Veldhuizen RAW, Thibodeaux BA, O’Callaghan RJ, Wright JR. 2005. Pseudomonas aeruginosa protease IV degrades surfactant proteins and inhibits surfactant host defense and biophysical functions. Am J Physiol Lung Cell Mol Physiol 288:L409–18. doi:10.1152/ajplung.00322.2004 15516485

[10] Bradshaw JL, Caballero AR, Bierdeman MA, Adams KV, Pipkins HR, Tang A, O’Callaghan RJ, McDaniel LS. 2018. Pseudomonas aeruginosa protease IV exacerbates pneumococcal pneumonia and systemic disease. mSphere 3:e00212-18. doi:10.1128/mSphere.00212-18 29720526 PMC5932373

[11] Barbier M, Damron FH, Bielecki P, Suárez-Diez M, Puchałka J, Albertí S, Dos Santos VM, Goldberg JB. 2014. From the environment to the host: re-wiring of the transcriptome of Pseudomonas aeruginosa from 22°C to 37°C. PLoS One 9:e89941. doi:10.1371/journal.pone.0089941 24587139 PMC3933690

[12] Wurtzel O, Yoder-Himes DR, Han K, Dandekar AA, Edelheit S, Greenberg EP, Sorek R, Lory S. 2012. The single-nucleotide resolution transcriptome of Pseudomonas aeruginosa grown in body temperature. PLOS Pathog 8:e1002945. doi:10.1371/journal.ppat.1002945 23028334 PMC3460626

[13] Robinson RE, Robertson JK, Prezioso SM, Goldberg JB. 2025. Temperature controls LasR regulation of piv expression in Pseudomonas aeruginosa. mBio 16:e00541-25. doi:10.1128/mbio.00541-25 40391957 PMC12153295

[14] Ménard G, Rouillon A, Cattoir V, Donnio P-Y. 2021. Galleria mellonella as a suitable model of bacterial infection: past, present and future. Front Cell Infect Microbiol 11:782733. doi:10.3389/fcimb.2021.782733 35004350 PMC8727906

[15] Andrejko M, Cytryńska M, Jakubowicz T. 2005. Apolipophorin III is a substrate for protease IV from Pseudomonas aeruginosa*.* FEMS Microbiol Lett 243:331–337. doi:10.1016/j.femsle.2004.12.024 15686832

[16] Andrejko M, Mizerska-Dudka M, Jakubowicz T. 2008. Changes in Galleria mellonella apolipophorin III level during Pseudomonas aeruginosa infection. J Invertebr Pathol 97:14–19. doi:10.1016/j.jip.2007.06.003 17681528

[17] Jander G, Rahme LG, Ausubel FM. 2000. Positive correlation between virulence of Pseudomonas aeruginosa mutants in mice and insects. J Bacteriol 182:3843–3845. doi:10.1128/JB.182.13.3843-3845.2000 10851003 PMC94559

[18] García-Reyes S, Moustafa DA, Attrée I, Goldberg JB, Quiroz-Morales SE, Soberón-Chávez G. 2021. Vfr or CyaB promote the expression of the pore-forming toxin exlBA operon in Pseudomonas aeruginosa ATCC 9027 without increasing its virulence in mice. Microbiology (Reading) 167. doi:10.1099/mic.0.001083 34424157

[19] Miyata S, Casey M, Frank DW, Ausubel FM, Drenkard E. 2003. Use of the Galleria mellonella caterpillar as a model host to study the role of the type III secretion system in Pseudomonas aeruginosa pathogenesis. Infect Immun 71:2404–2413. doi:10.1128/IAI.71.5.2404-2413.2003 12704110 PMC153283

[20] Almblad H, Randall TE, Liu F, Leblanc K, Groves RA, Kittichotirat W, Winsor GL, Fournier N, Au E, Groizeleau J, et al.. 2021. Bacterial cyclic diguanylate signaling networks sense temperature. Nat Commun 12:1986. doi:10.1038/s41467-021-22176-2 33790266 PMC8012707

[21] Ramarao N, Nielsen-Leroux C, Lereclus D. 2012. The insect Galleria mellonella as a powerful infection model to investigate bacterial pathogenesis. J Vis Exp 4392:e4392. doi:10.3791/4392 PMC3567165 23271509

[22] Joyce SA, Gahan CGM. 2010. Molecular pathogenesis of Listeria monocytogenes in the alternative model host Galleria mellonella*.* Microbiology (Reading) 156:3456–3468. doi:10.1099/mic.0.040782-0 20688820

[23] Chen W, Zhang Y, Zhang Y, Pi Y, Gu T, Song L, Wang Y, Ji Q. 2018. CRISPR/Cas9-based genome editing in Pseudomonas aeruginosa and cytidine deaminase-mediated base editing in Pseudomonas species. iScience 6:222–231. doi:10.1016/j.isci.2018.07.024 30240613 PMC6137401

[24] Gibson DG, Young L, Chuang R-Y, Venter JC, Hutchison CA, Smith HO. 2009. Enzymatic assembly of DNA molecules up to several hundred kilobases. Nat Methods 6:343–345. doi:10.1038/nmeth.1318 19363495

[25] Engler C, Gruetzner R, Kandzia R, Marillonnet S. 2009. Golden gate shuffling: a one-pot DNA shuffling method based on type IIs restriction enzymes. PLOS ONE 4:e5553. doi:10.1371/journal.pone.0005553 19436741 PMC2677662

[26] Traidej M, Caballero AR, Marquart ME, Thibodeaux BA, O’Callaghan RJ. 2003. Molecular analysis of Pseudomonas aeruginosa protease IV expressed in Pseudomonas putida*.* Invest Ophthalmol Vis Sci 44:190–196. doi:10.1167/iovs.02-0458 12506074

[27] Oh J, Li X-H, Kim S-K, Lee J-H. 2017. Post-secretional activation of Protease IV by quorum sensing in Pseudomonas aeruginosa*.* Sci Rep 7:4416. doi:10.1038/s41598-017-03733-6 28667333 PMC5493658

[28] Park S-J, Kim S-K, So Y-I, Park H-Y, Li X-H, Yeom DH, Lee M-N, Lee B-L, Lee J-H. 2014. Protease IV, a quorum sensing-dependent protease of Pseudomonas aeruginosa modulates insect innate immunity. Mol Microbiol 94:1298–1314. doi:10.1111/mmi.12830 25315216

[29] Jarrell KF, Kropinski AM. 1982. The virulence of protease and cell surface mutants of Pseudomonas aeruginosa for the larvae of Galleria mellonella*.* J Invertebr Pathol 39:395–400. doi:10.1016/0022-2011(82)90065-9 6123536

