# Supplementary material for: piv does not impact Pseudomonas aeruginosa virulence in Galleria mellonella
Source: Microbiol Spectr. 2025 May 21;13(7):e02811-24. doi: 10.1128/spectrum.02811-24 (PMC12210898; doi:10.1128/spectrum.02811-24)
Supplement: Supplemental material — Figures S1 and S2. [file spectrum.02811-24-s0001.docx]

**SUPPLEMENTAL MATERIALS**

*piv* does not impact *Pseudomonas aeruginosa* virulence in *Galleria mellonella*

Rachel E. Robinson^a,b^, Joshua K. Robertson^c^, Dina A. Moustafa^b,d^, and Joanna B. Goldberg^b,d#^

^a^Microbiology and Molecular Genetics Program, Graduate Division of Biological and Biomedical Sciences, Laney Graduate School, Emory University, Atlanta, Georgia, USA

^b^Department of Pediatrics, Division of Pulmonary, Asthma, Cystic Fibrosis, and Sleep, Emory University School of Medicine, Atlanta, Georgia, USA

^c^Department of Biology, Emory University, Atlanta, Georgia, USA

^d^Emory+Children's Center for Cystic Fibrosis and Airway Disease Research, Emory University School of Medicine, Atlanta, Georgia, USA

Running title: PIV does not impact virulence in *G. mellonella*

^#^Address correspondence to Joanna B. Goldberg, joanna.goldberg@emory.edu.

**Supplemental Figures**

**Figure S1**


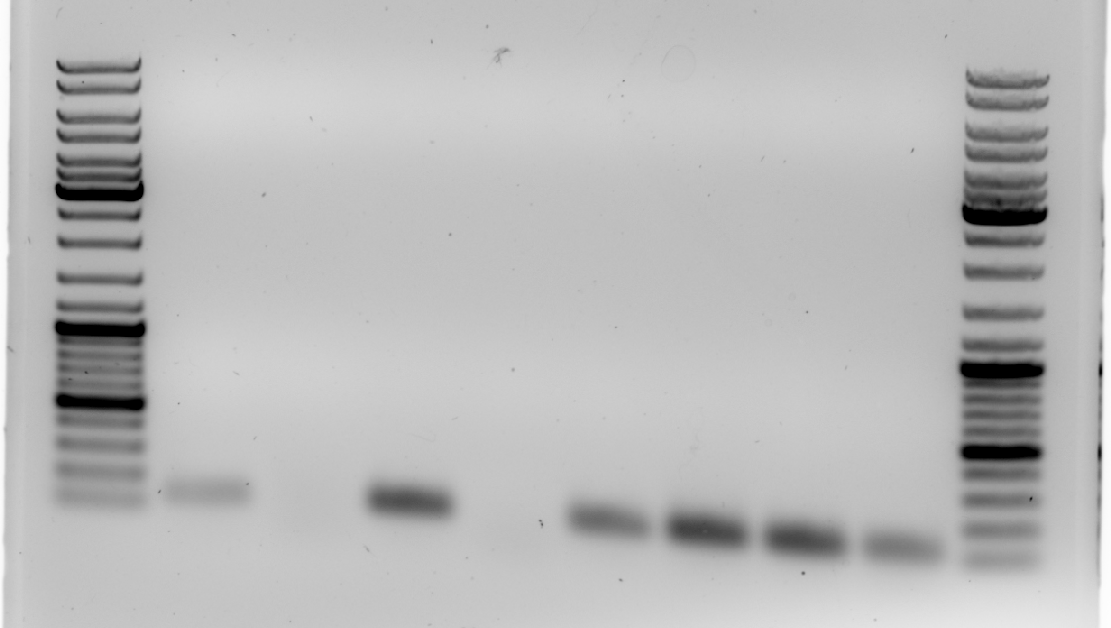


**1**

**2**

**3**

**4**

**5**

**6**

**7**

**8**

**9**

**10**

***mvaU***

***piv***

**Fig. S1.** RNA was extracted from hemolymph of 3-5 *G. mellonella* larvae infected with ~100 CFU of PAO1 and Δ*piv* at 25°C and 37°C as described and DNase treated prior to RT-qPCR with primers for *piv* and for *mvaU*, a housekeeping gene, whose expression does not vary with temperature. RT-qPCR products were visualized by gel electrophoresis to determine the presence or absence of *piv* transcripts during *G. mellonella* infection. RT-qPCR products for *piv* (lanes 2-5) or *mvaU* (lanes 6-9) from each group are as follows: 1 – 10 kb ladder, 2 – PAO1 at 37°C, 3 - Δ*piv* at 37°C, 4 – PAO1 at 25°C, 5 - Δ*piv* at 25°C, 6 – PAO1 at 37°C, 7 - Δ*piv* at 37°C, 8 – PAO1 at 25°C, 9 - Δ*piv* at 25°C, 10 – 10 kb ladder.

**Figure S2**

**A**


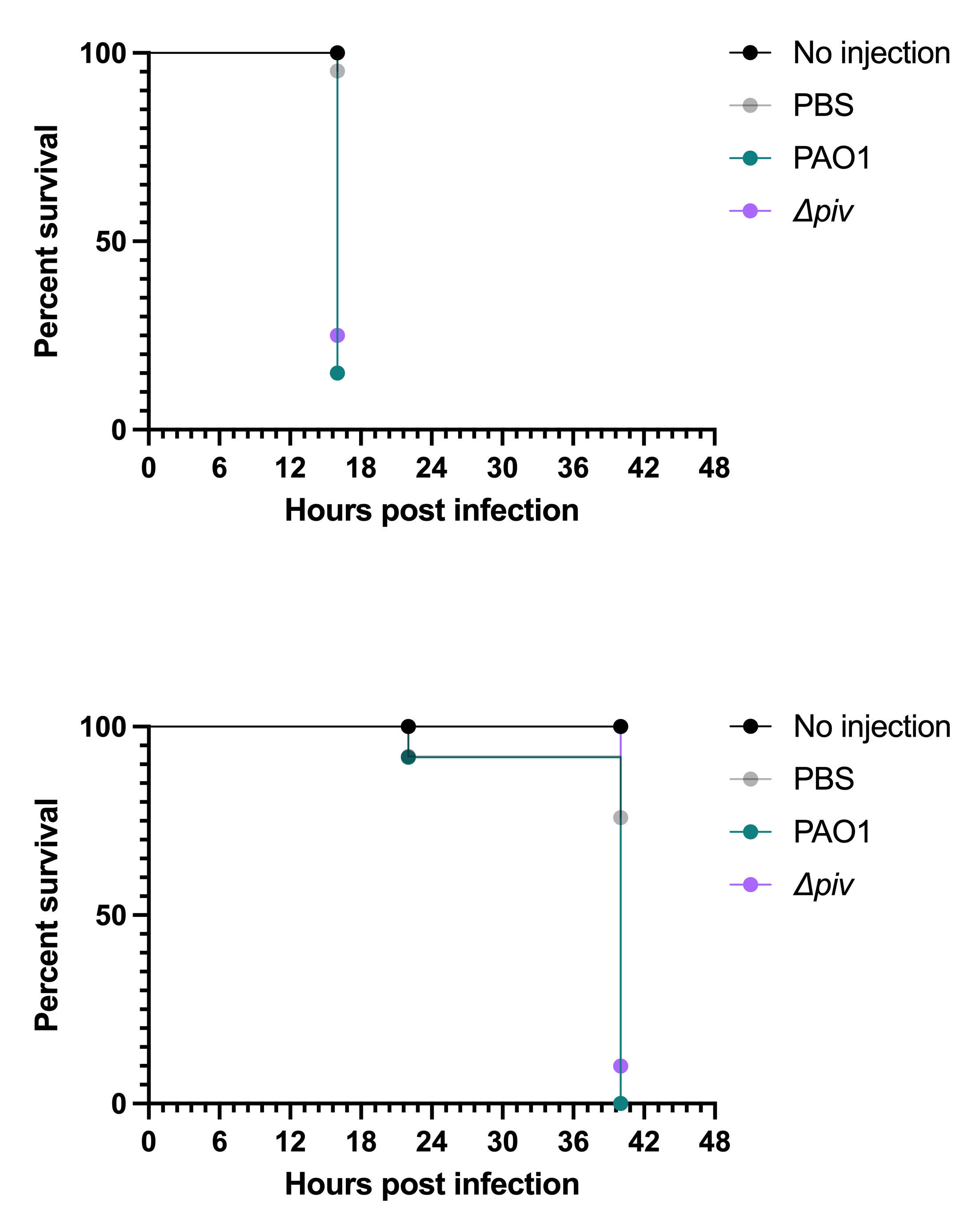


**B**

**Fig. S2.** *G. mellonella* larvae were infected with ~10 CFU of PAO1 and Δ*piv* from exponential phase cultures grown at 37°C (A) or 25°C (B) and then housed at the corresponding temperature as depicted in Figure 2. Larvae were also injected with an equal volume PBS or not injected as negative controls. 40 larvae were infected for each PAO1 and Δ*piv* groups, 21 for the PBS group, and 22 for the no infection group at each temperature. Kaplan-Meier survival curves represent the combined data from three independent experiments. Statistical significance was determined by log-rank test.
